# Synergistic Impact of Alpha-Fetoprotein and Tumor Burden on Long-Term Outcomes Following Curative-Intent Resection of Hepatocellular Carcinoma

**DOI:** 10.3390/cancers13040747

**Published:** 2021-02-11

**Authors:** Diamantis I. Tsilimigras, J. Madison Hyer, Adrian Diaz, Fabio Bagante, Francesca Ratti, Hugo P. Marques, Olivier Soubrane, Vincent Lam, George A. Poultsides, Irinel Popescu, Sorin Alexandrescu, Guillaume Martel, Aklile Workneh, Alfredo Guglielmi, Tom Hugh, Luca Aldrighetti, Itaru Endo, Timothy M. Pawlik

**Affiliations:** 1 Department of Surgery, The Ohio State University Wexner Medical Center, Columbus, OH 43210, USA; diamantis.tsilimigras@osumc.edu (D.I.T.); madison.hyer@osumc.edu (J.M.H.); adriandi@med.umich.edu (A.D.); fabio.bagante@gmail.com (F.B.); 2 Department of Surgery, University of Verona, 37134 Verona, Italy; alfredo.guglielmi@univr.it; 3 Department of Surgery, Ospedale San Raffaele, 20132 Milano, Italy; francesca.ratti@hsr.it (F.R.); aldrighetti.luca@hsr.it (L.A.); 4 Department of Surgery, Curry Cabral Hospital, 1069-166 Lisbon, Portugal; hugoscpm@gmail.com; 5 Department of Hepatobiliopancreatic Surgery, APHP, Beaujon Hospital, 92110 Clichy, France; olivier.soubrane@gmail.com; 6 Department of Surgery, Westmead Hospital, Sydney 2145, Australia; vincent.lam@sydney.edu.au; 7 Department of Surgery, Stanford University, Stanford, CA 94305, USA; gpoultsides@stanford.edu; 8 Department of Surgery, Fundeni Clinical Institute, 022328 Bucharest, Romania; irinel.popescu220@gmail.com (I.P.); stalexandrescu@yahoo.com (S.A.); 9 Department of Surgery, University of Ottawa, Ottawa, ON K1H 8M5, Canada; gumartel@toh.ca (G.M.); aworkneh@ohri.ca (A.W.); 10 Department of Surgery, School of Medicine, The University of Sydney, Sydney 2006, Australia; thugh@med.usyd.edu.au; 11 Department of Gastroenterological Surgery, Yokohama City University School of Medicine, Yokohama 236-0004, Japan; endoit@yokohama-cu.ac.jp

**Keywords:** tumor burden, AFP, HCC, resection, surgery

## Abstract

**Simple Summary:**

Hepatocellular carcinoma (HCC) tumor burden score (TBS) and α-fetoprotein (AFP) have been considered important predictors of outcomes among patients with resectable HCC; yet, the interplay of TBS (i.e., tumor morphology) and AFP (i.e., surrogate for tumor biology) in HCC has not been examined to date. The current study aimed to investigate the interplay of HCC TBS and AFP among patients undergoing resection for HCC. Both TBS and serum AFP levels were strong predictors of outcomes and demonstrated a synergistic impact on prognosis, with higher serum AFP predicting worse outcomes among patients with HCC of a certain TBS class after resection. Both tumor morphology (i.e., tumor burden) and tumor-specific biomarkers (i.e., serum AFP) may be important when assessing the prognosis of patients who undergo resection for HCC.

**Abstract:**

Introduction: The prognostic role of tumor burden score (TBS) relative to pre-operative α -fetoprotein (AFP) levels among patients undergoing curative-intent resection of HCC has not been examined. Methods: Patients who underwent curative-intent resection of HCC between 2000 and 2017 were identified from a multi-institutional database. The impact of TBS on overall survival (OS) and cumulative recurrence relative to serum AFP levels was assessed. Results: Among 898 patients, 233 (25.9%) patients had low TBS, 572 (63.7%) had medium TBS and 93 (10.4%) had high TBS. Both TBS (5-year OS; low TBS: 76.9%, medium TBS: 60.9%, high TBS: 39.1%) and AFP (>400 ng/mL vs. <400 ng/mL: 48.5% vs. 66.1%) were strong predictors of outcomes (both *p* < 0.001). Lower TBS was associated with better OS among patients with both low (5-year OS, low–medium TBS: 68.0% vs. high TBS: 47.7%, *p* < 0.001) and high AFP levels (5-year OS, low–medium TBS: 53.7% vs. high TBS: not reached, *p* < 0.001). Patients with low–medium TBS/high AFP had worse OS compared with individuals with low–medium TBS/low AFP (5-year OS, 53.7% vs. 68.0%, *p* = 0.003). Similarly, patients with high TBS/high AFP had worse outcomes compared with patients with high TBS/low AFP (5-year OS, not reached vs. 47.7%, *p* = 0.015). Patients with high TBS/low AFP and low TBS/high AFP had comparable outcomes (5-year OS, 47.7% vs. 53.7%, *p* = 0.24). The positive predictive value of certain TBS groups relative to the risk of early recurrence and 5-year mortality after HCC resection increased with higher AFP levels. Conclusion: Both TBS and serum AFP were important predictors of prognosis among patients with resectable HCC. Serum AFP and TBS had a synergistic impact on prognosis following HCC resection with higher serum AFP predicting worse outcomes among patients with HCC of a certain TBS class.

## 1. Introduction

Hepatocellular carcinoma (HCC) accounts for approximately 75% of all primary liver malignancies and represents the sixth most common cancer worldwide [1,2]. In the United States, HCC is the leading cause of cancer-related deaths, with epidemiologic data predicting a continued increase in the incidence of HCC until at least 2030 [1,2]. Surgical resection is the cornerstone of treatment and potentially curative treatment among patients with resectable HCC [3,4]. Nevertheless, the efficacy of surgical resection is limited by the relatively high rates of recurrence in the early (i.e., ≤2 years) or late (i.e., >2 years) postoperative period [5,6]. As such, there is a need for better risk stratification schemas to improve patient selection and optimize long-term outcomes following HCC resection.

Traditional predictors of outcomes among patients with resectable HCC have included morphologic (i.e., maximum tumor size and nodularity, collectively known as “tumor burden”) and pathologic (i.e., tumor grade, microvascular invasion, liver capsule invasion, etc.) characteristics, as well as serum biomarkers (i.e., α-fetoprotein (AFP), albumin, bilirubin etc.). Among these factors, tumor burden has been demonstrated to be important in driving prognosis and determining stage, as noted in both the American Joint Committee on Cancer (AJCC) 8th edition and the Barcelona Clinic Liver Cancer (BCLC) staging systems [7,8]. Apart from tumor morphology, serum AFP is another well-established predictor, with several studies highlighting the importance of this marker among patients with HCC [8,9]. Indeed, high AFP levels indicate an increase in tumor aggressiveness and may, in turn, predict inferior outcomes after both liver resection and transplantation for HCC [9,10].

Although traditional staging systems have largely used arbitrary cut-offs of tumor size and number to define staging [7,8], our group has proposed an alternative method to estimate tumor burden—the tumor burden score (TBS)—that incorporates both tumor size and number in a continuous fashion using the Pythagorean theorem (α^2^ + β^2^ = γ^2^, where α = maximum tumor diameter, β = number of lesions and γ = TBS) [11]. Of note, TBS demonstrated an excellent prognostic discrimination and dictated prognosis among patients with resectable HCC [11]. Similar to the “metro-ticket” approach in liver transplantation, however, the prognostic impact of TBS may vary depending on pre-operative AFP levels [12]. To date, the relative impact of TBS and AFP on outcomes among patients undergoing resection of HCC has not been examined. As such, the objective of the current study was to define the prognostic impact of TBS relative to pre-operative AFP levels among patients undergoing curative-intent resection of HCC. Specifically, we aimed to examine whether TBS had a varied impact on prognosis among patients with low versus high pre-operative AFP levels.

## 2. Methods

### 2.1. Patient Population

Patients who underwent hepatectomy for HCC between 2000 and 2017 were identified from an international multi-institutional database. Patients were treated at one of 11 participating institutions that comprised the International Hepatocellular Carcinoma Study Group [5,13]. Patients who did not receive curative-intent resection, had BCLC-C tumors (i.e., major vascular invasion or extrahepatic spread), did not have information on pathologic tumor size and number, did not have information on pre-operative AFP levels, or had inadequate follow-up data were excluded from the analysis. The study was approved by the Institutional Review Boards of all participating institutions.

### 2.2. Variables of Interest and Outcomes

Demographic and clinical data included age, sex, American Society of Anesthesiologists (ASA) performance score, history of cirrhosis, history of hepatitis B virus (HBV)/hepatitis C virus (HCV) infection, pre-operative alpha-fetoprotein (AFP), extent of surgical resection (i.e., minor or major), type of operative approach (i.e., minimally invasive surgery (MIS) or open), pathologic tumor size and number, TBS [11], tumor differentiation grade, presence of lympho-vascular invasion, liver capsule involvement, and margin status (i.e., R0, R1). Following liver resection, patients were followed for recurrence with serum AFP and imaging studies, including ultrasonography, computed tomography and/or magnetic resonance imaging once every 3–4 months for the first 3 years, once every 6 months from years 4–5 and then annually [13].

The primary outcome was overall survival (OS), defined as the time interval between liver resection and death or last follow-up. The secondary outcome was cumulative recurrence, defined as suspicious/positive findings on imaging or histologically confirmed disease during surveillance, as previously reported [5]. Early recurrence was defined as recurrence within 2 years following curative-intent resection of HCC [5]. The primary independent variables were pathologic TBS and pre-operative AFP levels. TBS was defined as the distance from the origin of a Cartesian plane and comprised of maximum tumor size (x-axis) and number of tumors (y-axis) so that TBS^2^ = (maximum tumor diameter)^2^ + (number of tumors)^2^, as previously described [11]. Based on previously reported cut-offs of TBS, [11] patients were categorized as having low (TBS ≤ 3.36), medium (TBS 3.36–13.74) or high TBS (TBS ≥ 13.74) [11]. In addition, patients were categorized as having low (i.e., <400 ng/mL) or high (i.e., >400 ng/mL) AFP, as previously reported [11,13,14]. For the analysis of TBS and AFP relative to long-term outcomes, patients with low and medium TBS were grouped together.

### 2.3. Statistical Analysis

Continuous variables were presented as median (interquartile range (IQR)) and categorical variables as frequency (%). The Kruskal–Wallis one-way analysis of variance was used to compare continuous variables, while categorical variables were compared using the chi-squared test or Fisher’s exact test, as appropriate. Differences in survival on bivariate levels were visualized using Kaplan–Meier curves and assessed using the log-rank test. The association of AFP and TBS with OS was assessed using a Cox regression analysis. Variables significant on bivariate analysis (*p*-value < 0.05) were entered into the multivariable model. The proportional hazards assumption was assessed using Schoenfeld residuals. The positive predictive value (PPV) for various combinations of AFP levels (i.e., <400 ng/mL vs. >400 ng/mL) and TBS (i.e., low, medium, high) relative to the occurrence of early recurrence and 5-year all-cause mortality after HCC resection was calculated. Statistical significance was assessed at α = 0.05. The level of statistical significance for all tests was set at α = 0.05. All statistical analyses were performed with the SPSS, v26 (IBM Corp., Armonk, NY, USA) and JMP, v14 (SAS Institute Inc., Cary, NC, USA) statistical packages.

## 3. Results

### 3.1. Baseline Characteristics of Patients

A total of 898 patients underwent curative-intent resection for HCC and were included in the final analytic cohort. Most patients were male (n = 680, 75.8%) with a median age of 67 years (IQR: 59–74) and had an ASA score of ≤2 (n = 521, 64.4%). Approximately 40% of patients (n = 353, 39.4%) had cirrhosis, while history of HBV and HCV infection was present in 27.7% (n = 246) and 32.7% (n = 290) of patients, respectively. The majority of patients had pre-operative AFP levels <400 ng/mL (n = 725, 80.7%). The median tumor size was 4.8 cm (IQR: 3.0–8.5) and median TBS was 5.1 (3.4–8.6). A total of 233 (25.9%) patients had low TBS, 572 (63.7%) had medium TBS and 93 (10.4%) had high TBS. Approximately one-third of patients underwent major liver resection (n = 309, 35.3%). On pathology, 38.5% (n = 311) of patients had lympho-vascular invasion and 32.8% (n = 224) of patients had liver capsule involvement. The vast majority of patients underwent an R0 resection (n = 779, 88.9%) (Table 1).

### 3.2. Impact of TBS and AFP on OS and Recurrence Rates

At a median follow-up of 28.5 months (IQR: 12.9–52.7), 5-year OS following HCC resection was 62.7% in the entire cohort. Both TBS and AFP were strong predictors of outcomes when examined separately. In particular, 5-year OS incrementally worsened with higher TBS (low TBS: 76.9%, medium TBS: 60.9%, high TBS: 39.1%; *p* < 0.001). In addition, patients with high AFP (i.e., AFP > 400 ng/mL) had worse 5-year OS compared with patients who had low AFP (i.e., AFP < 400 ng/mL) (48.5% vs. 66.1%; *p* < 0.001). Similarly, a higher TBS was associated with higher cumulative recurrence rates following HCC resection (low vs. medium vs. high TBS, 1-year recurrence: 19.2% vs. 27.6% vs. 48.2%; 3-year recurrence: 41.1% vs. 56.2% vs. 68.7%; *p* < 0.001), as were the higher pre-operative AFP levels (low vs. high AFP; 1-year recurrence: 22.5% vs. 47.3%; 3-year recurrence: 49.3% vs. 65.3%; *p* < 0.001).

**Figure 1:**
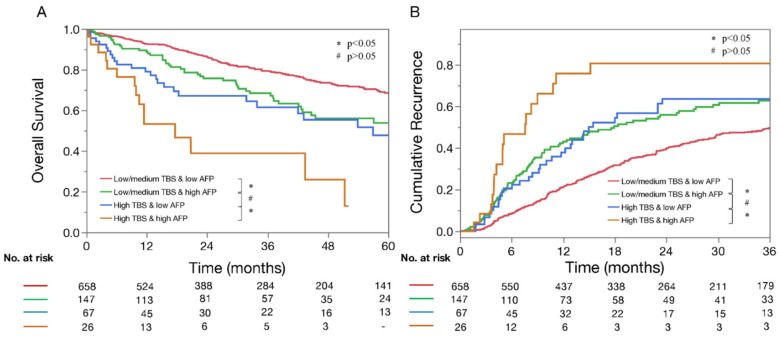
Kaplan–Meier curves demonstrating differences in OS (**A**) and recurrence (**B**) among patients with low–medium tumor burden score (TBS)/low a-fetoprotein (AFP), low–medium TBS/high AFP, high TBS/low AFP and high TBS/high AFP. * *p* < 0.05, # *p* > 0.05.

In examining combinations of TBS and AFP relative to long-term outcomes, lower TBS was strongly associated with better OS irrespective of low (5-year OS, low–medium TBS: 68.0% vs. high TBS: 47.7%, *p* < 0.001) or high AFP levels (5-year OS, low–medium TBS: 53.7% vs. high TBS: not reached; *p* < 0.001) (Figure 1A). Similarly, lower TBS was associated with lower 3-year recurrence rates among patients with both low (low–medium TBS: 49.5% vs. high TBS: 63.5; *p* = 0.02) and high AFP levels (low–medium TBS: 62.7% vs. high TBS: 80.6%; *p* = 0.01) (Figure 1B).

### 3.3. Synergistic Impact of TBS and AFP on Early Recurrence and 5-Year Death

After combining TBS and AFP levels, four distinct groups were generated: low–medium TBS/low AFP (n = 658, 73.3%), low–medium TBS/high AFP (n = 147, 16.4%), high TBS/low AFP (n = 67, 7.4%) and high TBS/high AFP (n = 26, 2.9%). Of note, patients with high TBS/high AFP were older (median age: 69 years (IQR: 62–77)) and more often had lympho-vascular invasion (n = 22, 84.6%) and poor to undifferentiated tumors (n = 12, 46.2%) (all *p* < 0.05). In contrast, patients with low–medium TBS/low AFP were younger (median age: 60 years (IQR: 67–74), more frequently had a history of cirrhosis (n = 287, 43.8%) and had undergone MIS resection (n = 174, 26.4%) (all *p* < 0.05). History of HBV and HCV infection was more prevalent among patients with low–medium TBS irrespective of AFP levels (Table 2, both *p* < 0.001). R0 resections were achieved more frequently among patients with low AFP (low–medium TBS/low AFP: 90.3%; high TBS/low AFP: 90.9%; low–medium TBS/high AFP: 81.9%; high TBS/high AFP: 88.5%; *p* = 0.03) (Table 2).

**Figure 2:**
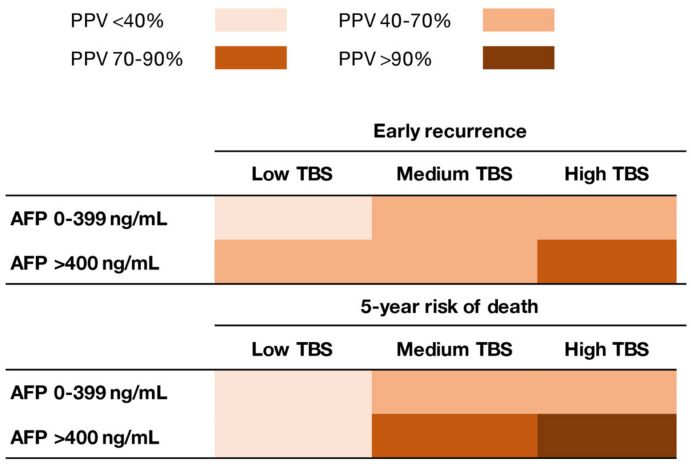
Heat map demonstrating differences in the positive predictive value (PPV) of TBS and AFP levels relative to early recurrence and 5-year mortality after resection for hepatocellular carcinoma (HCC).

**Figure 3:**
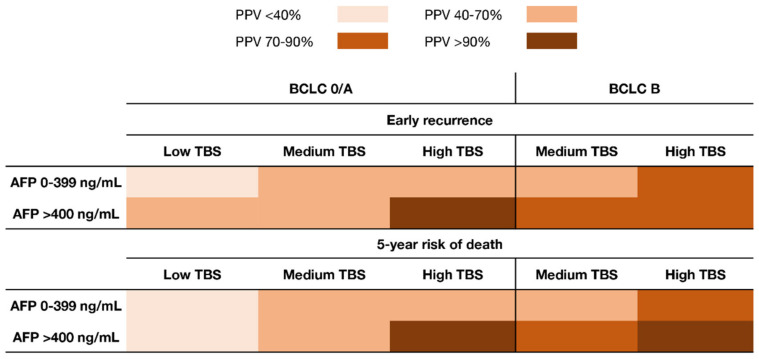
Heat map demonstrating differences in the PPV of TBS and AFP levels relative to early recurrence and 5-year mortality after resection for HCC stratified by BCLC-0/A and BCLC-B stage.

Of note, TBS and AFP demonstrated a synergistic impact on outcomes. In particular, the impact of TBS on outcomes varied depending on serum AFP levels. Patients with low–medium TBS/high AFP had worse OS compared with individuals with low–medium TBS/low AFP (5-year OS, 53.7% vs. 68.0%; *p* = 0.003). Similarly, patients with high TBS/high AFP had worse outcomes compared with patients with high TBS/low AFP (5-year OS, not reached vs. 47.7%; *p* = 0.015). Of note, patients with high TBS/low AFP had comparable outcomes as patients with low TBS/high AFP (5-year OS, 47.7% vs. 53.7%; *p* = 0.24) (Figure 1). When assessing the risk of early recurrence (i.e., recurrence within 2 years after resection), the PPV of TBS increased with higher AFP levels (low AFP; low TBS: 36.7%, medium TBS: 49.0%, high TBS: 67.3%; high AFP: low TBS: 55.0%, medium TBS: 62.7%, high TBS: 89.5%). Similarly, when assessing the risk of 5-year death following HCC resection, the PPV of TBS increased with higher AFP levels (low AFP; low TBS: 39.4%, medium TBS: 52.7%, high TBS: 67.6%; high AFP; low TBS: 36.4%, medium TBS: 73.3%, high TBS: 100%) (Figure 2). Of note, the same associations were noted among patients with both BCLC-0/A and BCLC-B HCC (Figure 3).

On multivariable analysis, after adjusting for all competing factors, patients with high TBS/low AFP had 63% (HR = 1.63, 95%CI 1.03–2.56) higher hazards of death compared with individuals who had low–medium TBS/low AFP. Of note, patients with high TBS/high AFP had markedly higher hazards of death (HR = 3.66, 95%CI 2.03–6.58) compared with individuals with low–medium TBS/low AFP (Table 3).

## 4. Discussion

Tumor burden has been considered a major predictor of outcomes and an important element in the treatment decision-making process of patients with resectable HCC [11,15]. Our group recently demonstrated the prognostic utility of TBS, a relatively new marker of tumor burden, which dictated prognosis among patients with resectable HCC [11]. Whether the impact of TBS varied according to serum AFP levels had, however, not been previously examined. The current study built on the previous work as it analyzed the cumulative impact of TBS (i.e., metric of tumor morphology) and serum AFP levels (i.e., metric of tumor aggressiveness) among patients with resectable HCC. Both TBS and serum AFP levels were strong predictors of long-term outcomes after resection of HCC. Of note, TBS was able to predict prognosis among patients with either low (i.e., less aggressive) or high (i.e., more aggressive) AFP levels. Importantly, TBS and AFP also demonstrated a synergistic impact on outcomes. Specifically, patients with similar TBS had varied outcomes depending on serum AFP levels, with the worst outcomes noted among patients with high TBS/high AFP. After adjusting for other competing risk factors, the combination of low/high TBS and low/high AFP remained an independent predictor of OS among patients undergoing resection for HCC.

Traditional assessment of tumor burden in HCC has used a binary categorization of tumor size and number as noted in the most recent AJCC and BCLC staging systems [7,8]. More recently, investigators have utilized a continuum of tumor size and number as a means to enhance the prognostication of patients with HCC [11,16]. Although originally developed to predict prognosis after resection for colorectal liver metastases [17], TBS was recently used to predict prognosis among patients with resectable HCC and demonstrated excellent performance [11]. TBS was further able to stratify prognosis within certain BCLC stages, suggesting that this metric may be a valuable tool to estimate prognosis of patients undergoing resection for HCC [11]. Apart from tumor morphology, however, serum AFP has been widely recognized as a surrogate of tumor biology and is considered a strong predictor of outcomes among patients with HCC [18,19]. Serum AFP is a well-established marker of tumor aggressiveness and has been used in clinical practice to screen high-risk patients, as well as in informing prognoses following primary HCC treatment [18,19]. In turn, although the utility of TBS has been independently evaluated using a large international multi-institutional dataset [11], whether TBS has a varied impact on long-term outcomes according to serum AFP levels warranted further investigation.

As such, the present study examined the inter-play of serum AFP (i.e., tumor biology) and HCC TBS (i.e., tumor morphology) by investigating four groups of patients: low–medium TBS/low AFP, low–medium TBS/high AFP, high TBS/low AFP and high TBS/high AFP (Table 2). Patients with high TBS/high AFP more frequently had lympho-vascular invasion and poor-to-undifferentiated tumors, which had previously been associated with unfavorable outcomes [5,9,11]. Of note, lymphovascular invasion and tumor differentiation can only be ascertained after resection and, thus, serum AFP may act as a surrogate of these unfavorable characteristics in the pre-operative setting [9,10]. In addition, only a minority of patients with high TBS/high AFP had underlying cirrhosis (7.7%), while a significantly higher proportion (43.8%) of patients with low–medium TBS/low AFP had a cirrhotic liver. The reasons for these differences may be due to the need for better underlying liver function among patients with larger tumor burdens to be considered candidates for surgical resection in order to minimize the chance of post-hepatectomy liver failure [20]. Interestingly, patients with high TBS were older than individuals with low–medium TBS, which may suggest a more indolent course of HCC among elderly patients that resulted in disease identification at a more advanced stage than younger patients [21]. Of note, age per se is not a contraindication to surgery, as selected elderly patients may benefit from hepatectomy for HCC [22]. Perhaps not surprisingly, only a minority of patients with high TBS/high AFP underwent resection via a MIS approach (3.8%), which was in contrast to the higher percentage noted among patients with low–medium TBS (low AFP: 26.4%, high AFP: 20.8%). Collectively, the data strongly suggested that the clinical presentation and characteristics of patients with HCC varied significantly according to TBS and serum AFP.

Incorporating tumor-specific biomarkers to prognostic HCC schemas may enhance the predictive accuracy and, in turn, maximize clinical benefit through optimization of patient selection for surgical resection [8,23]. The French Liver Transplantation Study Group suggested incorporating AFP to tumor size and number to better identify appropriate candidates for liver transplantation [24]. The “AFP model” was superior to the traditional Milan criteria (i.e., morphologic-only criteria) in predicting HCC recurrence after liver transplantation and was adopted in 2013 by the French organization for Organ Sharing to select transplant candidates [24]. In line with these findings, the current study demonstrated that both TBS (i.e., tumor morphology) and AFP played a significant role in predicting outcomes after HCC resection. Of note, TBS and AFP had a synergistic impact on patient outcomes; in particular, patients with low–medium TBS/high AFP had worse OS compared with individuals with low–medium TBS/low AFP (5-year OS, 53.7% vs. 68.0%; *p* = 0.003). Similarly, although OS was generally poor among patients with high TBS, having high AFP was associated with an even worse prognosis (5-year OS, high TBS/high AFP: not reached vs. high TBS/low AFO: 47.7%; *p* = 0.015, Figure 1). Interestingly, individuals with high TBS/low AFP had comparable outcomes to patients with low–medium TBS/high AFP, which suggested that serum AFP levels contributed to a “stage migration effect” when assessing the impact of TBS on long-term prognosis.

Apart from tumor morphology and AFP, the liver tumor microenvironment also determines tumor biology and aggressiveness. Indeed, an increase in the number of regulatory T cells, tumor-associated macrophages and myeloid-derived suppressor cells in HCC has been associated with tumor progression and poor prognosis [25]. In addition, accumulating evidence suggests that cancer progression is closely correlated with the host immune response and immune cell infiltrates. In other words, there is a vicious cycle between cancer cells and the immune microenvironment that may determine outcomes [26]. To this end, investigators have proposed an alternative score, the “immuno-score” that calculates CD3+ and CD8+ T cells in the center and invasive margin of the tumor, which may further stratify outcomes of patients with HCC [25,26]. Although the predictive ability of this score has been validated in a number of malignancies [25,27], information on tumor cell infiltration was not available in the current dataset. In turn, further analysis on the association of the immuno-score and the combination of TBS/AFP relative to patient outcomes was not possible in this study.

Another interesting finding of the current study was that the combination of high TBS/high AFP was associated with an excellent PPV of >90% relative to death within 5 years after HCC resection; in contrast, the PPV associated with high TBS/low AFP was much lower (40%–70%) (Figure 2). While patients with high TBS comprised only about 10% of the cohort, similar findings were noted among patients with medium TBS who comprised the majority of the study sample (63.7%). Of note, among patients with medium TBS and low AFP, the PPV of TBS relative to a 5-year death after resection was 40%–70%, which increased to more than 70% among individuals with high AFP (Figure 2). After stratifying by the BCLC stage, patients with BCLC-B HCC with medium TBS and low AFP had a similar risk of early recurrence and 5-year all-cause mortality versus individuals with BCLC-0/A HCC (Figure 3); these data suggested that this subset of BCLC-B patients benefited from resection beyond the current BCLC resection criteria [11,28,29,30,31]. Ding et al. had reported that an AFP score ≤ 2 identified a subgroup of BCLC-B/C patients with generally favorable outcomes after resection of HCC [9]. Collectively, the data from the current study highlight the importance of both tumor morphology and serum AFP levels to predict outcomes of patients undergoing resection of HCC. In addition, the data suggested that surgeons should consider pre-operative AFP levels to optimize patient selection, especially when considering a major resection for high tumor burden disease.

Certain limitations should be taken into account when interpreting the results of this study. Owing to the retrospective nature of this work, selection bias regarding which patients were offered surgery (i.e., patients with high TBS likely had a more favorable tumor profile and were fit for surgery) was possible. In addition, while the same surveillance protocol was in place for all participating institutions, any deviation from these protocols may have impacted the detection of recurrence. Furthermore, while the multi-institutional nature of the study was a strength, there might be variations in patient selection and surgical techniques among participating centers that may have influenced the results. The present study only analyzed patients with BCLC-0, A and B HCC and, thus, the data may not be applicable to patients with more advanced (i.e., BCLC-C) or unresectable HCC. Since the current study analyzed a surgical cohort (i.e., a portion of the overall population of HCC patients), this cohort may not be representative of the overall patient population with HCC. In turn, the association of TBS and AFP in different populations (i.e., non-surgical populations, patients unfit for surgery etc.) could potentially vary, thus no conclusions can be drawn for non-surgical patients from the results of the current study. Rather, given the multi-institutional nature of the study, this cohort is representative of the surgical patients presenting with resectable HCC worldwide.

## 5. Conslusions

In conclusion, both TBS and serum AFP were important predictors of prognosis among patients with resectable HCC. Serum AFP and TBS had a synergistic impact on prognosis following HCC resection with higher serum AFP predicting worse outcomes among patients with HCC of a certain TBS class. Data from the current retrospective study highlight that both tumor morphology (i.e., tumor burden) and tumor-specific biomarkers (i.e., serum AFP) could be important when assessing the prognosis of patients who undergo resection for HCC. Further large-scale trials are needed to validate the results of the current study among patients with both resectable and unresectable disease.

## Figures and Tables

**Table 1:** Clinicopathologic characteristic of the entire cohort.

Variables	Total (n = 898)
Age †	67 (59–74)
Sex	
Male	680 (75.8%)
Female	218 (24.2%)
ASA-PS	
≤2	521 (64.4%)
>2	288 (35.6%)
Cirrhosis	353 (39.4%)
HBV infection	
No	642 (72.3%)
Yes	246 (27.7%)
HCV infection	
No	598 (67.3%)
Yes	290 (32.7%)
AFP, ng/mL	
≤400	725 (80.7%)
>400	173 (19.3%)
Minimally Invasive Surgery	211 (23.6%)
Type of resection	
Minor	566 (64.7%)
Major	309 (35.3%)
Tumor size of largest nodule, cm †	4.8 (3.0–8.5)
Tumor number †	1 (1–1)
Tumor Burden Score †	5.1 (3.4–8.6)
Low	233 (25.9%)
Medium	572 (63.7%)
High	93 (10.4%)
BCLC stage	
BCLC-0	59 (6.6%)
BCLC-A	690 (76.8%)
BCLC-B	149 (16.6%)
Grade	
Well to moderate	679 (78.6%)
Poor to undifferentiated	185 (21.4%)
Lympho-vascular invasion	
No	496 (61.5%)
Yes	311 (38.5%)
Liver capsule involvement	
No	458 (67.2%)
Yes	224 (32.8%)
Margin Status	
R0	779 (88.9%)
R1	97 (11.1%)

† median (IQR) IQR = interquartile range; ASA-PS = American Society of Anesthesiologists-Performance score; HBV = hepatitis B virus; HCV = hepatitis C virus; AFP = α-fetoprotein; BCLC = Barcelona Clinic Liver Cancer.

**Table 2:** Characteristics of patients stratified by TBS and AFP levels.

Variables	Low/Medium TBS and AFP < 400 ng/mL (N = 658, 73.3%)	Low/Medium TBS and AFP > 400 ng/mL (N = 147, 16.4%)	High TBS and AFP < 400 ng/mL (N = 67, 7.4%)	High TBS and AFP > 400 ng/mL (N = 26, 2.9%)	*p*-Value
Age †	60 (67–74)	65 (56–71)	69 (62–76)	69 (62–77)	**0.03**
Sex					0.27
Male	500 (76.1%)	104 (70.7%)	55 (82.1%)	21 (80.8%)	
Female	157 (23.9%)	43 (29.3%)	12 (17.9%)	5 (19.2%)	
ASA-PS					0.56
≤2	381 (63.8%)	89 (69.0%)	37 (63.8%)	14 (56.0%)	
>2	216 (36.2%)	40 (31.0%)	21 (36.2%)	11 (44.0%)	
Cirrhosis	287 (43.8%)	55 (37.4%)	9 (13.4%)	2 (7.7%)	**<0.001**
HBV infection					**<0.001**
No	472 (72.7%)	91 (61.9%)	59 (89.4%)	20 (76.9%)	
Yes	177 (27.3%)	56 (38.1%)	7 (10.6%)	6 (23.1%)	
HCV infection					**<0.001**
No	414 (63.6%)	96 (66.2%)	64 (97.0%)	24 (92.3%)	
Yes	237 (36.4%)	49 (33.8%)	2 (3.0%)	2 (7.7%)	
Minimally Invasive Surgery	174 (26.4%)	30 (20.8%)	6 (9.1%)	1 (3.8%)	**0.001**
Type of resection					**<0.001**
Minor	472 (73.9%)	74 (51.4%)	15 (22.4%)	5 (20.0%)	
Major	167 (26.1%)	70 (48.6%)	52 (77.6%)	20 (80.0%)	
Grade					**<0.001**
Well to moderate	536 (85.1%)	83 (58.5%)	46 (69.7%)	14 (53.8%)	
Poor to undifferentiated	94 (14.9%)	59 (41.5%)	20 (30.3%)	12 (46.2%)	
Lympho-vascular invasion	172 (29.2%)	80 (62.5%)	37 (58.7%)	22 (84.6%)	**<0.001**
Liver capsule involvement	151 (30.4%)	47 (43.5%)	18 (32.7%)	8 (36.4%)	0.07
Margin Status					**0.03**
R0	578 (90.3%)	118 (81.9%)	60 (90.9%)	23 (88.5%)	
R1	62 (9.7%)	26 (18.1%)	6 (9.1%)	3 (11.5%)	

† median (IQR) IQR = interquartile range; ASA-PS: American Society of Anesthesiologists-Performance score; HBV = hepatitis B virus; HCV = hepatitis C virus; AFP = α-fetoprotein; TBS = tumor burden score. Bold values denote statistical significance

**Table 3:** Cox regression analysis of factors associated with overall survival.

Variable	Bivariate Analysis		Multivariable Analysis	
	HR (95% CI)	*p*-Value	HR (95% CI)	*p*-Value
Age	1.01 (0.99–1.02)	0.14		
Gender (male)	1.05 (0.79–1.40)	0.74		
ASA-PS (III–IV)	1.25 (0.94–1.66)	0.12		
Liver cirrhosis	1.15 (0.89–1.48)	0.27		
MIS	0.55 (0.38–0.81)	**0.002**	0.66 (0.45–0.98)	**0.04**
Major resection	1.55 (1.20–2.00)	**0.001**	1.09 (0.81–1.45)	0.58
Grade (poor/undifferentiated)	1.70 (1.28–2.24)	**<0.001**	1.49 (1.11–1.99)	**0.007**
Lympho-vascular invasion	1.90 (1.45–2.48)	**<0.001**	1.33 (0.97–1.83)	0.08
Liver capsule involvement	1.37 (1.04–1.82)	**0.02**	1.31 (0.94–1.82)	0.11
R1 margins	1.51 (1.04–2.20)	**0.03**	1.31 (0.89–1.92)	0.18
Group				
Low/Medium TBS and low AFP	Ref		Ref	
Low/Medium TBS and high AFP	1.59 (1.16–2.18)	**0.004**	1.23 (0.87–1.73)	0.24
High TBS and low AFP	2.14 (1.42–3.22)	**<0.001**	1.63 (1.03–2.56)	**0.03**
High TBS and high AFP	5.28 (3.09–9.03)	**<0.001**	3.66 (2.03–6.58)	**<0.001**

ASA-PS = American Society of Anesthesiologists-Performance score; MIS = minimally invasive surgery; AFP = alpha-fetoprotein; TBS = tumor burden score. Bold values denote statistical significance.

## Data Availability

The data presented in this study are available upon request from the corresponding author.
